# Statistical approaches to identifying significant differences in predictive performance between machine learning and classical statistical models for survival data

**DOI:** 10.1371/journal.pone.0279435

**Published:** 2022-12-28

**Authors:** Justine B. Nasejje, Albert Whata, Charles Chimedza

**Affiliations:** 1 School of Statistics and Actuarial Science, University of the Witwatersrand, Johannesburg, Gauteng, South Africa; 2 School of Natural and Applied Sciences, Sol Plaatje University, Kimberley, Northern Cape, South Africa; Mustansiriyah University - College of Science, IRAQ

## Abstract

Research that seeks to compare two predictive models requires a thorough statistical approach to draw valid inferences about comparisons between the performance of the two models. Researchers present estimates of model performance with little evidence on whether they reflect true differences in model performance. In this study, we apply two statistical tests, that is, the *5 × 2*-fold cv paired *t*-test, and the combined *5 × 2*-fold cv *F*-test to provide statistical evidence on differences in predictive performance between the Fine-Gray (FG) and random survival forest (RSF) models for competing risks. These models are trained on different scenarios of low-dimensional simulated survival data to determine whether the differences in their predictive performance that exist are indeed significant. Each simulation was repeated one hundred times on ten different seeds. The results indicate that the RSF model is superior in predictive performance in the presence of complex relationships (quadratic and interactions) between the outcome and its predictors. The two statistical tests show that the differences in performance are significant in quadratic simulation but not significant in interaction simulations. The study has also revealed that the FG model is superior in predictive performance in linear simulations and its differences in predictive performance compared to the RSF model are significant. The combined *5 × 2*-fold cv *F*-test has lower type I error rates compared to the *5 × 2*-fold cv paired *t*-test.

## Introduction

The advent of machine learning has provided challenges especially to the statistical community [1]. Unlike the classical statistical models that have decision theory embedded in them, the machine learning models are yet to have this theory embedded within them. The fears of over fitting, type I and II errors have therefore led to criticism of machine learning models since they were first conceived [2].

The statistical community has for sometime ignored these models until it became categorically clear that they can not be ignored especially in this era of big data. There has therefore been a shift in research on making sure that robust tests are developed to make sure that the machine learning and statistical models agree at least on the basics or the building blocks of statistical theory [3].

In this study, we focus on statistical tests to evaluate whether the difference in the predictive performance of the Fine-gray (FG) [4] and the random survival forests (RSF) [5] models for competing risks data are significant under three low-dimensional data simulation scenarios. Both the FG and the RSF models for competing risk outcomes, model time-to-event distributions for mutually exclusive event.

In the analysis of time-to-event outcomes, a competing risk is an event whose occurrence precludes the occurrence of the event of primary interest [6]. This complicates the analysis of such a dataset [6–8].

When outcomes are time-to-event in nature, the objective of prognostic models is frequently focused on estimating the cumulative incidence function (CIF) [7].

The cause-specific hazard approach is the most commonly used classical statistical approach in analysing competing risk data [4]. However, it treats events other than the event of interest as censored. This leads to inflated survival probabilities and therefore does not result into meaningful conclusions [9, 10]. The Fine-Gray model or the proportional hazards model for the sub-distribution approach is known to handle competing risks well by allowing the events that are competing with the event of interest to continue being in the risk set [4]. The Fine-gray model also has an advantage of directly modeling the effects of the covariates on the cumulative incidence function [10]. An alternative state of the art model in modelling competing risk events is the random survival forest for competing risks [5]. It is a machine learning model whose goal is to also estimate the CIF. Assessing the accuracy of predictions from the above mentioned models is an important part in their development. This is because they are commonly used in predicting important and very sensitive biological phenomena of occurrence of binary outcomes like presence of disease, death within a given duration of time, or hospital readmission within a given duration of time [11]. A study by [12] noted that methods for assessing the calibration of prognostic models for use with competing risk data have received little attention.

A recent study by [7] provides strong evidence that random survival forests models predict default and prepayment risk more accurately than statistical benchmarks in the form of the Cox proportional hazard model and the Fine and Gray model. However, no statistical tests were used to show evidence for the significance difference in their predictive performance.

Properties of the random survival model in modeling competing risks in low and high dimension data were studied by [5]. The authors’ results show that the Fine-gray model was better than the random survival forest model in predictive performance in linear low-dimension settings. The results further show that that the random survival forest is better in non-linear low-dimension settings. To obtain these results, they compared the predictive performance values of the models with all the covariates to a benchmark or threshold value. The threshold model’s predictive performance value was obtained from the null model that ignored all the covariates. In this study, we use two statistical tests to evaluate whether the difference in the predictive performance of the Fine-gray and the random survival forests models for competing risks data is significant under three low-dimensional data simulation scenarios.

We employed two statistical tests, namely; the *5 × 2*-fold cv paired *t*-test, and the combined *5 × 2*-fold cv *F*-test [13, 14] via a simulation study to examine whether the differences in the predictive performance of the two models are significant in each of the three scenarios considered.

The rest of the article is structured as follows: Section 2, describes the nature of competing risks data and the methods used in this study; Section 3, describes the two statistical tests; Section 4, describes the simulation study; Section 5, presents the simulation results, and in Section 6, we discuss and present conclusions of the study.

## Competing risk models in survival analysis

A competing risk is an event that, if it occurs, prevents the primary event of interest from occurring. For competing risks, we are interested in the time *T*_*j*_ between the time origin and the occurrence of an event of interest. Individuals who are subjected to competing risks are observed from the time they enter the study to the time the competing event or the event of interest occurs. Often, individuals are observed before the occurrence of one of the events. To describe the nature of competing risk data let $T_{j}^{0}$ denote event time for the *j*^*th*^ individual, and let $\delta_{j}^{0}$ be his or her event type, such that $\delta_{j}^{0}\in \left\{1\ldots K\right\}$, where *K* ≥ 1. Furthermore, we let $C_{j}^{0}$ denote the individual’s censoring time such that the actual time of event $T_{j}^{0}$ is unobserved and one only observes $T_{j}=\text{min}(T_{j}^{0},C_{j}^{0})$ and the event indicator $\delta_{j}=\delta_{j}^{0}I(T_{j}^{0}\leq C_{j}^{0})$. When *δ*_*j*_ = 0, the individual is said to be censored at *T*_*j*_, otherwise if *δ*_*j*_ = *k* > 0, the individual is said to have an event of type *k* at time *T*_*j*_. Thus, the observed competing risk data is such that (*T*_*j*_, *δ*_*j*_, *X*_*j*_)_1≤*j*≤*n*_ where *X*_*j*_ is a *p*-dimensional vector of covariates. In addition, we let *t*_1_ < *t*_2_ < … < *t*_*m*_, *m* ≤ *n*, be distinct event times.

Thus, the main goal of survival analysis is to estimate the survival probability of the event *T*_*j*_ for a new instance using the feature predictors denoted by *X*_*j*_. It should be noted that in survival analysis problems, *T*_*j*_ will be both continuous and non-negative.

## The survival and hazard functions

The survival function *S*(*t*) is represented by:

$$\begin{array}{c} S(t)=P(T\geq t)\;. \end{array}$$ (1)

Eq 1 estimates the probability that the survival probability of an event of interest does not occur before time *t* [15, 16]. *S*(*t*) is non-negative and has an initial condition, (*S*(0) = 1), indicating that 100% of the observed individuals survive when none of the events of interest has occurred. The survival function has two important properties: *S*(0) = 1 (i.e., the event has not yet occurred for any subjects at the start of the study) and lim_*t*→∞_*S*(*t*) = 0 (i.e., the event of interest eventually occurs for all subjects).

The hazard function (λ(*t*)), is another commonly used function that is referred to as the *instantaneous death rate* [17].

The hazard function is mathematically defined by [16]:

$$\begin{array}{cc} \lambda (t) & =\underset{\Delta t\to 0}{\text{lim}}\frac{P\{t\leq T\leq t+\Delta t|T\geq t\}}{\Delta t}\;,\\ & =\underset{\Delta t\to 0}{\text{lim}}\frac{F(t+\Delta t)-F\left(t\right)}{\Delta t.S(t)}\;,\\ & =\frac{f(t)}{S(t)}\;. \end{array}$$ (2)

λ(*t*), is a non-negative function. According to [18], the survival function *S*(*t*) can also be expressed as:

$$\begin{array}{c} S\left(t\right)=e^{-\Lambda (t)}\;, \end{array}$$ (3)

where $\Lambda \left(t\right)=\int_{0}^{t}\lambda \left(u\right)du$ is the *Cumulative Hazard Function (CHF)*.

## Survival probability prediction

### Fine Gray model

The survival function S(t) can be estimated using traditional statistical methods and machine learning methods. This study focuses on a semi-parametric method, the Fine-Gray (FG) [4] model to evaluate the cumulative incidence function (CIF). Fine and Gray [4] developed the sub-distribution hazard function defined by;

$$\begin{array}{c} \lambda_{k}^{*}(t)=\underset{\Delta t\to 0}{\text{lim}}\frac{P(t\leq T\leq t+\Delta t,\delta =k|T\geq t\cup (T<t\cap \delta \neq k\cap C^{0}>t))}{\Delta t}\;. \end{array}$$ (4)

where $\lambda_{k}^{*}(t)$ is known as the sub-distribution hazard and it measures the instantaneous rate of occurrence of the event of interest among subjects that have not yet experienced it. In this study,

$$\delta_{j}\in \left\{1,2\right\}\;,$$

and our interest is modeling the cumulative incidence function for failure from cause 1 conditional on the covariates.

As reported by [19], classical survival methods are not appropriate to analyse time-to-event data in complex situations such as in a competing risk setup, in which an individual in the risk set is exposed to multiple causes of failure. The proportional hazard (PH) model [20] is one of the classical methods for analysing competing risk data to examine the effect of covariates on the cause specific hazard function. The main drawback of using the PH model in a competing risk setup is that when estimating regression parameters for a specific cause, it considers individuals failing for reasons other than the cause of interest as censored observations [19, 21]. To address the limitation of the PH model, Fine and Gray [4] developed a survival regression based model that uses the cumulative incidence function (CIF) and sub-distribution hazard functions to describe the likelihood of an event occurring prior to a specific time. Unlike the PH model, the CIF does not exclude other competing risks when a specific cause is of interest [22].

The cumulative incidence function (CIF) is defined by *CIF*(*k*) = *P*(*T* ≤ *t*, *δ*_*j*_ = *k*).

Furthermore, CIF(k) represents the probability of the *k*^*th*^ event occurring before time *t* and before the occurrence of another type of event [21]. This means that CIF allows for the estimation of the occurrence of an event while accounting for competing risk. A key point is that, in the competing risks setting, only one event type can occur, such that the occurrence of one event precludes the subsequent occurrence of other event types.

Although the FG model was developed to address the limitations of Cox-based models, there is still considerable confusion regarding how the estimates from FG models are interpreted [23]. The confusion arises because the regression coefficients associated with this model are unclear or incorrectly interpreted. Also, when comparing results from different studies, an incorrect and inconsistent interpretation of the regression coefficients can cause confusion. Furthermore, an incorrect interpretation of the estimated regression coefficients can lead to an incorrect understanding of the magnitude of the relationship between exposure and incidence of the outcome.

The predictive performance of FG, a classical statistical model, is compared to that of a machine learning model, the random survival model (RSF). When the PH assumption is violated, survival trees and random survival forests (RSF) approaches offer an appealing alternative to Cox proportional hazards models [24]. Survival trees and RSF extend the classification and regression trees [24]. In addition, survival tree methods are non-parametric, flexible, and capable of dealing with high-dimensional covariate data.

### Random survival forests for competing risks

**Fig 1:**
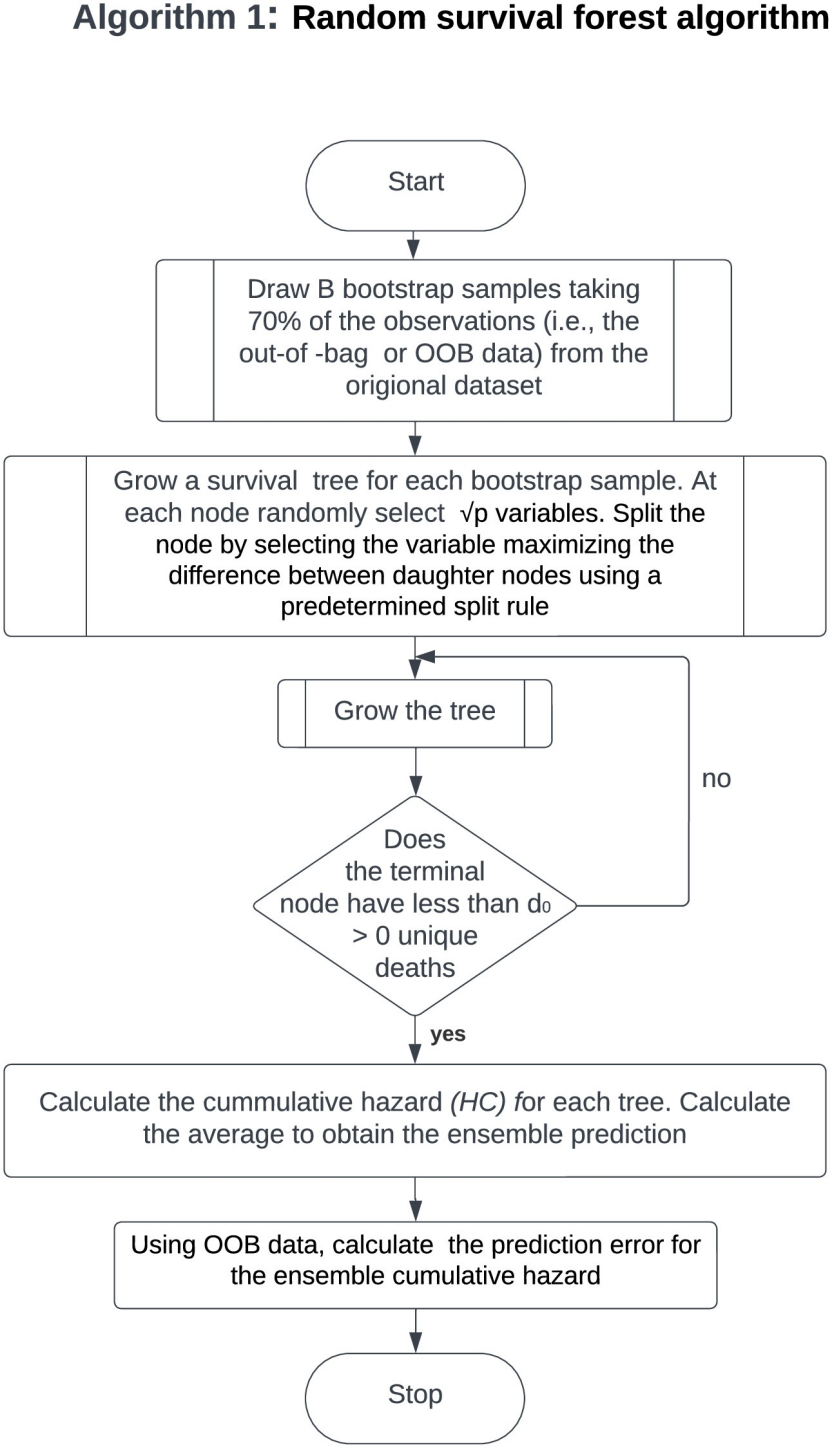
Algorithm 1: The flow chart illustrates the details of Algorithm 1, that is to say, the random survival forest algorithm. This a general algorithm for building a random survival forest. A survival tree is grown for each bootstrap sample by splitting the node after selecting a variable that maximizes the difference between daughter nodes using a predetermined split rule.

In recent years, random forests [25] have been extended to regression problems and survival outcomes. The random survival forest (RSF) algorithm [26] is a collection of survival trees that extends the random forest to evaluate survival analysis with censored data. The RSF’s algorithm implementation [26] is illustrated in a flowchart in Fig 1 below.

**Fig 2:**
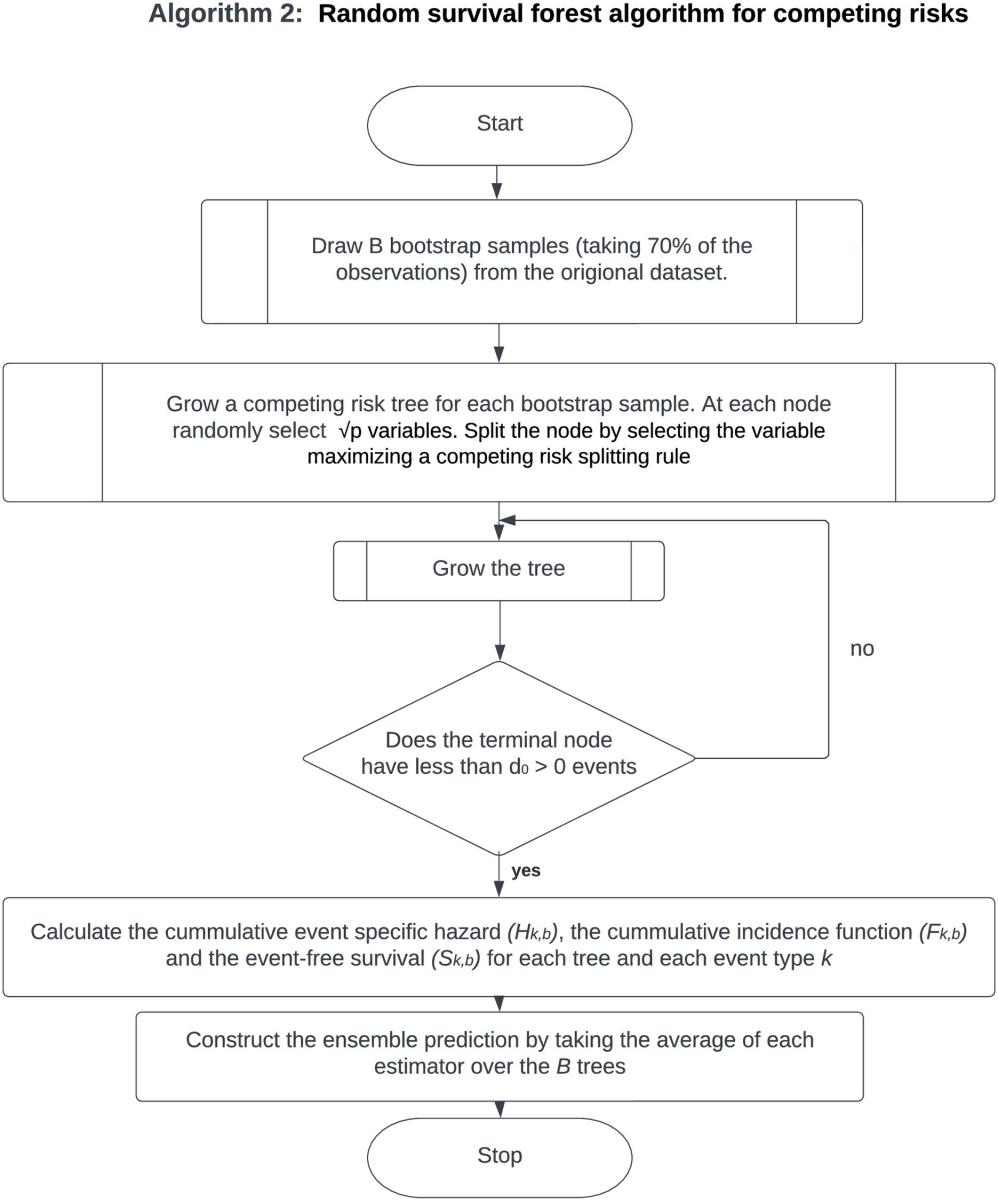
Algorithm 2: The flow chart illustrates the details of Algorithm 2, that is to say, the random survival forest algorithm for competing risks. The algorithm grows a competing risk tree for each bootstrap sample by Splitting the node after selecting a variable that maximizes the competing risk splitting rule.

RSFs have also been extended to competing risks. Random survival forests for competing risks are grown in a manner similar to the general algorithm (Algorithm 1) in Fig 1 of random survival forests, with the main difference being the splitting rule used [27, 28]. Furthermore, the RSF differs from the random forest method in that the RSF’s tree-growing splitting rule takes into account both the survival time and the censoring indicator. In this study, we will implement RSFs for competing risks Algorithm 2 outlined in the flowchart in Fig 2 that uses the log-rank splitting rule described in detail in [29] to split nodes by maximizing the log-rank test statistic. Before we outline the random survival forest algorithm for competing risks, we describe the split criteria used.

#### The generalised log-rank split-rule

Let the number of individuals at risk in the two daughter nodes be *R*_*α*_(*t*_*j*_) and *R*_*γ*_(*t*_*j*_), respectively. Then $R_{\alpha }(t_{j})=\sum_{j=1}^{n}I(T_{j}\geq t,x_{j}\leq s)$, $R_{\gamma }(t_{j})=\sum_{j=1}^{n}I(T_{j}\geq t,x_{j}>s)\;$, and *x*_*j*_ is the *x*-predictor for individual *j* = 1, 2, …, *n*. The total number of individuals at risk at time *t* is *R*(*t*) = *R*_*α*_(*t*) + *R*_*γ*_(*t*). The number of type *K* events for the left and right daughter nodes is, respectively.

$$\begin{array}{ccc} d_{k,\alpha }(t) & =\sum_{j=1}^{n}I(T_{j}=t,\delta_{j}=k,x_{j}\leq s)\;,\;d_{k,\gamma }(t) & =\sum_{j=1}^{n}I(T_{j}=t,\delta_{j}=k,x_{j}\;>s)\;, \end{array}$$ (5)

and *d*_*k*_(*t*) = *d*_*k*,*α*_(*t*) + *d*_*k*,*γ*_(*t*), is the number of type *k*, events at time *t*. Suppose that $\;t_{m}\;,t_{m_{\alpha }}\;,$ and $t_{m_{\gamma }}$ are the largest times of study in the root node and the two daughters, respectively. The generalised log-rank split-rule in the competing risk setting is based on a null hypothesis that the *H*_0_: λ_*k*,*α*_(*t*) = λ_*k*,*γ*_(*t*), ∀*t* ≤ *τ*, where *τ*, is a fixed time point set by the user in accordance with the observed follow-up period for the given dataset [5]. The split-rule at a point *s* on covariate *x* is given as:

$$\begin{array}{c} i_{k}(x,s)=\frac{1}{{\hat{\sigma }}_{k\;,\alpha }(x,s)}\sum_{j=1}^{m}W_{k}(t_{j})[d_{k\;,\alpha }(t_{j})-\frac{d_{k}R_{\alpha (t_{j})}}{R(t_{j})}]\;, \end{array}$$ (6)

where ${\hat{\sigma }}_{k\;,\alpha }(x,s)$ is the variance estimate given by:

$$\begin{array}{c} {\left[{\hat{\sigma }}_{k\;,\alpha }(x,s)\right]}^{2}=\sum_{j=1}^{m}W_{k}{\left(t_{j}\right)}^{2}d_{k}(t_{j})\frac{R_{\alpha }(t_{j})}{R(t_{j})}[1-\frac{R_{\alpha }(t_{j})}{R(t_{j})}][\frac{R(t_{j})-d_{k}(t_{j})}{R(t_{j})-1}]\;. \end{array}$$ (7)

Time-dependent weights, *W*_*k*_(*t*) > 0, are used to make the test more sensitive to early or late differences between the cause-specific hazards. The best split is found by maximizing, |*i*_*k*_(*x*, *s*)|, over all covariates and the split-points. Often the log-rank splitting rule is used to build trees for competing risks. As earlier stated, it tests the null hypothesis *H*_0_: λ_*k*,*α*_(*t*_*j*_) = λ_*k*,*γ*_(*t*_*j*_), ∀*t*_*j*_ ≤ *t*, which makes it inefficient in accounting for competing risks. It is therefore recommended that one uses the Gray’s test. An approximation to the Gray’s test which is performed by modifying the risk set of the log-rank test is available and implemented in R. It is a weighted log-rank test for testing the equivalence of the subdistribution hazard functions between two groups. It tests the null hypothesis *H*_0_: *F*_*k*,*α*_(*t*_*j*_) = *F*_*k*,*γ*_(*t*_*j*_), ∀*t*_*j*_ ≤ *t*

## Methods

### Simulations

#### Data simulations

We used the Cox-exponential cause-specific hazard approach [5, 30] to simulate competing risk data. This is the standard approach that is achieved by formulating competing risk data using the hazard for each cause:

$$\begin{array}{cc} \lambda_{k}(t|X) & =\lambda_{0k}\text{exp}(\beta_{k}^{T}X)\;, \end{array}$$ (8)

where λ_*k*_(*t*∣*X*) is the cause specific hazard for event *k* at time *t* for an individual with covariates *X*, λ_0*k*_ is a baseline hazard function that describes the risk for individuals with no covariate information, and $\text{exp}(\beta_{k}^{T}X)$ is the relative risk for two competing events *k* = 1, 2, given a vector of covariates *X* = (*x*_1_, *x*_2_, …, *x*_*p*_). With two competing risk events, the cause specific hazards of event one and two given the covariates are defined using:

$$\begin{array}{cc} & \lambda_{1}(t|X),\;\text{and}\;\lambda_{2}(t|X), \end{array}$$ (9)

where λ_1_(*t*|*X*) and λ_2_(*t*|*X*) are the cause specific hazards for event 1 and 2 at time *t*, respectively. The overall hazard is defined as:

$$\begin{array}{c} \lambda (t|X)=\lambda_{1}(t|X)+\lambda_{2}(t|X)\;. \end{array}$$ (10)

In all simulations, we set λ_0*k*_ = 0.01. Six continuous covariates (*x*_1_, *x*_2_, …, *x*_6_), were drawn independently from a standard normal distribution and six binary predictors (*x*_7_, *x*_8_, …, *x*_12_), from a binomial distribution with success probability of 50%. We considered the following three simulation scenarios for low-dimensional data (*p* < *n*):

i) Linear simulations;
ii) Quadratic simulations; and
iii) Interaction simulations.

#### Linear simulations

The linear simulation scenario has an additive structure, and we set the effect size of the covariates at:

$$\begin{array}{cc} \beta_{1} & =(a_{1},-a_{1},0,0,a_{1},-a_{1},a_{2},-a_{2},0,0,a_{2},a_{2})\;,\\ \beta_{2} & =(0,0,a_{1},-a_{1},a_{1},-a_{1},0,0,a_{2},-a_{2},a_{2},-a_{2})\;. \end{array}$$ (11)

The continuous effect size was set at *a*_1_ = log(2), and the discrete effect size was set at *a*_2_ = 1.5. Covariates *x*_1_, *x*_2_, *x*_7_, *x*_8_ have an effect on the hazard of event one only, whereas, covariates *x*_5_, *x*_6_, *x*_11_, *x*_12_ have an effect on both hazards. The covariates *x*_3_, *x*_4_, *x*_9_, *x*_10_ have an effect on the hazard of event two only.

#### Quadratic simulations

The linear additive structure was broken by introducing squared covariates $x_{1}^{2},x_{2}^{2},\ldots ,x_{6}^{2}$ with their effect sizes set at:

$$\begin{array}{cc} \beta_{1}^{Quad} & =(a_{1},-a_{1},0,0,-a_{1},a_{1})\;,\\ \beta_{2}^{Quad} & =(0,0,a_{1},-a_{1},a_{1},-a_{1})\;. \end{array}$$ (12)

#### Interaction simulations

The interaction terms are constructed as:

$$\begin{array}{c} \beta_{k}^{Int}I\left\{x_{l}>0\right\}x_{i}\;\;\text{for}\;\;l=\left\{1,2,\ldots ,6\right\}\;\;\text{and}\;\;i=\left\{7,8,\ldots ,12\right\}\;. \end{array}$$

The interaction effect sizes for the interaction terms are set at:

$$\begin{array}{cc} \beta_{1}^{Int} & =(-a_{1},a_{1},0,0,a_{1},-a_{1})\;,\\ \beta_{2}^{Int} & =(0,0,-a_{1},a_{1},-a_{1},a_{1})\;. \end{array}$$ (13)

## Experiments

### Model training for each simulation experiment

In this simulation experiment, we consider two models, the RSF and the FG models. The R packages *randomForestSRC* [31] and *cmprsk* [32] were used to implement the random survival forest for competing risks and the Fine-Gray model, respectively. Six datasets with sample sizes; 200, 300, 400, 500, 2000 and 3000 are used. For each simulation experiment the dataset is divided into two equal-sized sets, and the models are trained on one set and tested on the other. The difference between the error rates (integrated Brier scores) of the models are computed. The t-statistics, the F-statistics and the p-values associated with the tests are evaluated in each experiment. For the random survival forest, 500 trees are trained using the “logrankCR” splitting rule. A default terminal node size, *n*_0_ = 15 is used. Randomized splitting as described above is used, that is to say, at each parent node, for each of the randomly selected subset of covariates, “nsplit” randomly selected split points were chosen. The tree node is then split on that variable and random split point maximizing the absolute value of the split-statistic. For this simulation study, nsplit is set at 2 (nsplit = 2) because the simulation study has both continuous and discrete covariates. A small nsplit value is recommended in cases where there are both discrete and continuous covariates [5, 24]. The number of randomly selected subsets of the covariates to split on at each node known as “mtry” is set at $\;\sqrt{p}$.

### Model evaluation for each simulation experiment

#### Evaluation metrics

The integrated Brier score (IBS) [33] is used as a measure of predictive performance for both models. The IBS is the squared difference between actual and predicted outcome.

#### Integrated Brier score (IBS)

The Brier score is used when one is investigating the overall performance of survival models. It is desirable to have a model that is both discriminative (high concordance) and calibrated [34]. The Brier score is desirable because it measures both calibration and discrimination.

The Brier score is the average squared distances between the observed survival status and the predicted survival probability. For example, at a given time point *t*, the Brier score for a single subject is the squared difference between the observed event status (e.g., 1 = alive at time *t* and 0 = dead at time *t*) and a model based prediction of surviving to time *t*. For a test sample of size *n*_test_, the Brier score at time *t*, is given by:

$$\begin{array}{c} \begin{array}{cc} BS(t) & =\frac{1}{n_{\text{test}}}\sum_{l=1}^{n_{\text{test}}}\{{\left[0-\hat{S}(t|x)\right]}^{2}\frac{I(t_{l}\leq t,\delta_{l}=1)}{\hat{G}(t_{l}|x)}\\ & +{\left[1-\hat{S}(t|x)\right]}^{2}\frac{I(t_{l}>t)}{\hat{G}(t|x)}\}\;. \end{array} \end{array}$$ (14)

Where $\hat{G}(t|x)\approx P(C>t|X=x)$, is the Kaplan-Meier estimate of the conditional survival function of the censoring times. These are weightings of the Brier score to adjust for the presecnce of censored survival times. The integrated Brier score (*IBS*) is often used and it is given by:

$$\begin{array}{cc} IBS & =\int_{0}^{\text{max}(t)}BS(t)dt\;. \end{array}$$ (15)

The IBS gives an average Brier score across a time interval, and we use it as a metric to compare the performance of the FG and RSF models. As stated above, the Brier score is used to measure both calibration and discrimination. This implies that it can be employed when one is evaluating the overall performance of survival models or when the goal is to find a model that performs well on both calibration and discrimination.

The *5 × 2*-fold cv paired *t*-test, and the combined *5 × 2*-fold cv *F*-test statistics are calculated based on the differences of the values of IBS scores.

### Approximate statistical tests for comparing the Fine-Gray model and the random survival forest

Statistical hypothesis tests can be used to evaluate whether the difference in performance between two models is statistically significant. Two tests, that is, the *5 × 2*-fold cv paired *t*-test [13], and the combined *5 × 2*-fold cv *F*-test [14] were used in this study to determine whether the difference in the predictive performance between the FG and RSF models are significant.

#### *5 × 2*-fold cv paired *t*-test

The *K*-fold cross-validated paired t-test is the most commonly used method for comparing the performance of two models. The problem with this method, however is that the training sets overlap and it is therefore not recommended to be used in practice [13]. The *5 × 2*-fold cv paired *t*-test solves the problem of overlap in the training datasets that is prevalent in *K*-fold cross-validation paired *t*-test [13]. In addition, the *5 × 2*-fold cv paired *t*-test yields larger test data and training data sets that do not overlap. Thus, the *5 × 2*-fold cv paired *t*-test becomes a more powerful test compared to the *k*-fold cross-validated paired t-test. This is because it measures directly the variation that is brought about by the choice of the training data set. The *5 × 2*-fold cv paired *t*-test is therefore used as a post-hoc analysis to test whether the differences in the mean Brier scores of the FG and RSF models are statistically significant. The test statistic $\tilde{t}$, for the *5 × 2*-fold cv paired *t*-test is calculated as:

$$\begin{array}{c} \tilde{t}=\frac{p_{1}^{\left(1\right)}}{\sqrt{\frac{1}{5}\sum_{i=1}^{5}s_{i}^{2}}} \end{array}$$ (16)

where $p_{1}^{\left(1\right)}$ is the difference in the Brier Scores of the FG and RSF models for the first fold of the first iteration, $s_{i}^{2}$ is the variance of the Brier Scores differences of the *i*th iteration. The variance is computed using:

$$\begin{array}{cc} s_{i}^{2}= & {\left(p_{i}^{\left(1\right)}-\bar{p_{i}}\right)}^{2}+{\left(p_{i}^{\left(2\right)}-\bar{p_{i}}\right)}^{2}\;. \end{array}$$ (17)

In addition, $p_{i}^{\left(j\right)}$ is the difference in the Brier Scores of the FG and RSF models for the *i*^th^ iteration and fold *j*. Note that:

$$\begin{array}{cc} \bar{p_{i}} & =(p_{i}^{\left(1\right)}+p_{i}^{\left(2\right)})/2\;. \end{array}$$ (18)

**Fig 3:**
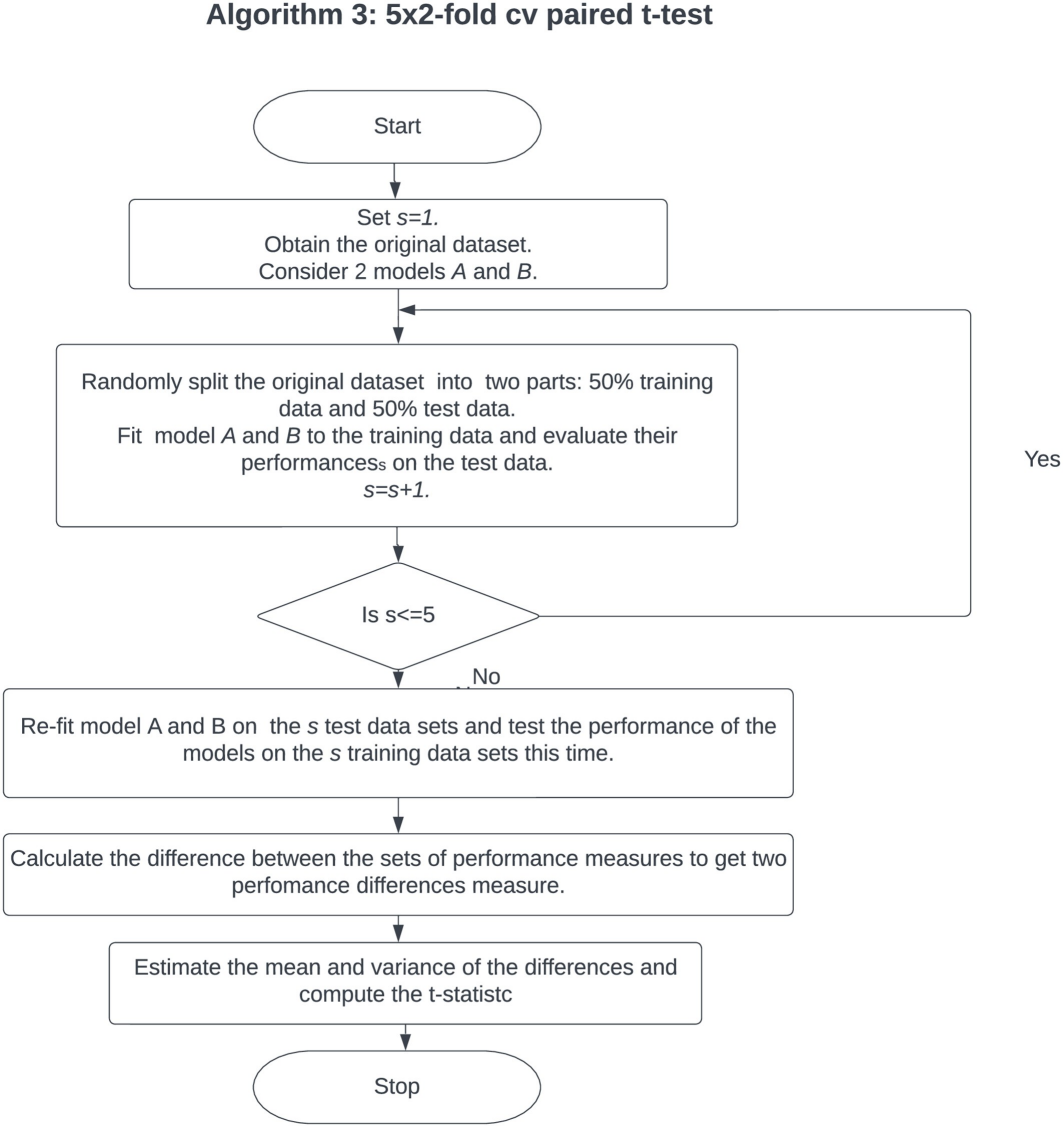
Algorithm 3: The flow chart illustrates the details of Algorithm 3, that is to say, *5 × 2*-fold cv paired *t*-test algorithm. The algorithm calculates the difference between two sets of performance measures by estimating the mean and variance of the differences and then computes the t-statistc.

The flowchart in Fig 3 below is Algorithm 3 for the *5 × 2*-fold cv paired *t*-test.

Although the *5 × 2*-fold cv paired t-test described in Fig 3 produces acceptable Type I errors, it fails in situations where performance metric’s scores that are measured in the various 2-fold cross-validation replications vary wildly [14].

#### Combined *5 × 2*-fold cv F-test

A study by [14] proposed a variant, the combined *5 × 2*-fold cv F test, that combines the results of the 10 possible statistics to get a more robust test. The test statistic of the combined 5 × 2-fold cv F-test is computed using:

$$\begin{array}{c} f=\frac{\sum_{i=1}^{5}\sum_{j=1}^{2}{\left(p_{i}^{\left(j\right)}\right)}^{2}}{2\sum_{i=1}^{5}s_{i}^{2}}\;. \end{array}$$ (19)

The statistic *f* is approximately *F* distributed with 10 and 5 degrees of freedom, and the hypothesis that the FG and RSF algorithms have the same value of the evaluation measurement is rejected if the statistic *f* is greater than 4.74 at *α*-level equal to 0.05. To compare the performance of FG and RSF models, the integrated Brier score (IBS) is used in this study.

#### Type I error

To control Type I error, that is, the likelihood of rejecting the null hypothesis that is true at some level *α*, we should reject the null hypothesis when the observed p-value is less than *α*:

$$\begin{array}{c} P_{H_{0}}\left(p-value\leq \alpha \right)=\alpha \;. \end{array}$$ (20)

The *p*-value is a random variable that depends on the observed data used to compute it. From the definition of the cumulative distribution function of any random variable, when the null is true, the *p*-value has a uniform distribution on the interval 0 ≤ *p*-value ≤ 1 klammer2009statistical. If the null is true, the sample of the *p*-values will look exactly like a sample of uniform random variables from the interval [0, 1]. To calculate the the Type I error of the *5 × 2*-fold cv paired *t*-test, and the combined *5 × 2*-fold cv *F*-test. The null and alternative hypotheses are such that:

**Hypothesis H_0_**: There is no significant difference in performance between the two models.
**Hypothesis H_1_**: There is a significant difference in performance between the two models.

## Results

The simulations were repeated 100 times at ten different seeds for each of the sample sizes considered in the study. For each sample size, there is therefore a total of 1000 independent simulations.

**Fig 4:**
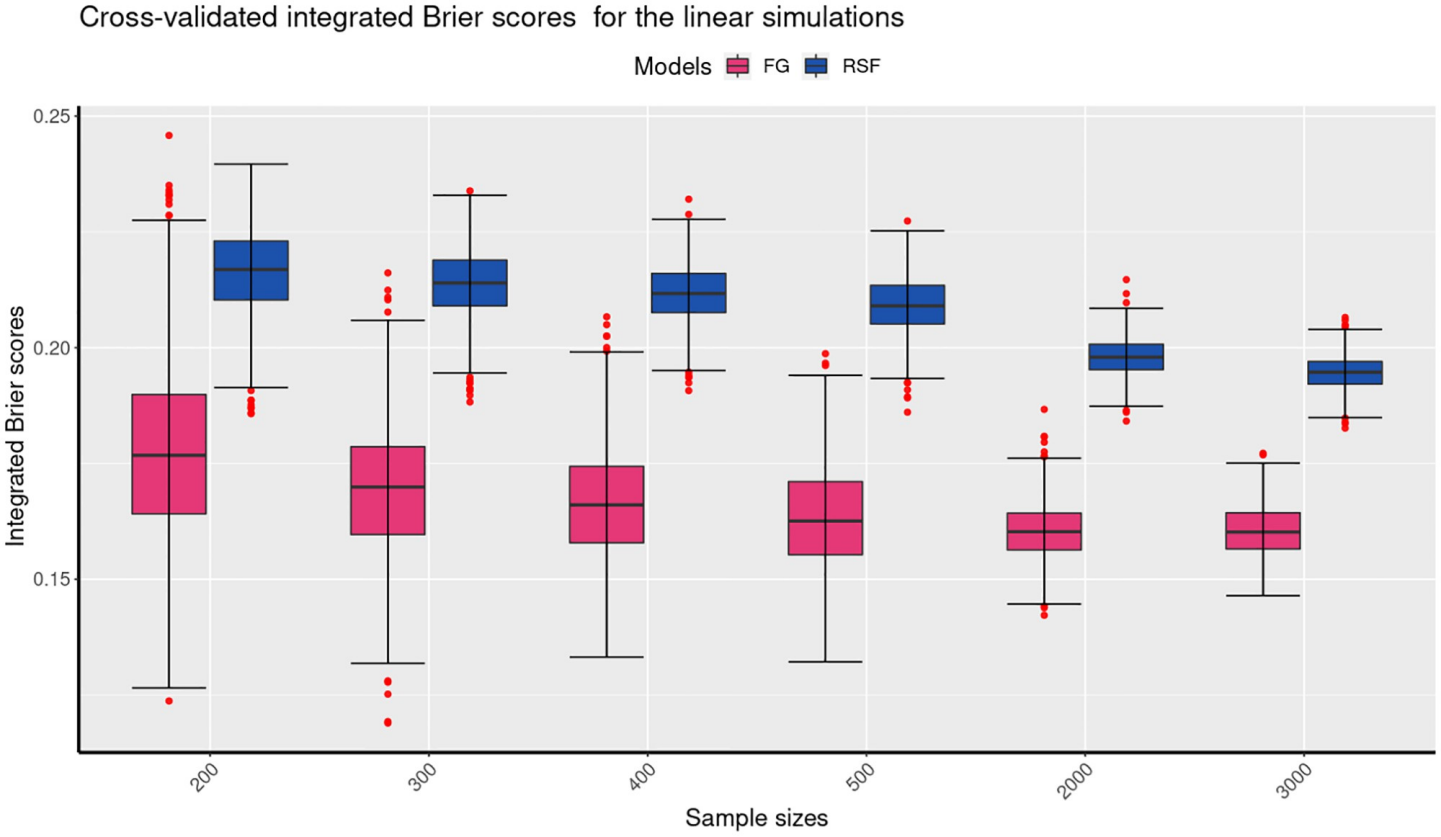
The boxplots present the mean cross-validated integrated brier scores from the 1000 simulations for each of the sample sizes for the linear simulations. The boxplots show the performance of the two models at six different sample sizes, 200, 300, 400, 500, 2000 and 3000. The mean IBS values show that for the linear simulations, the FG performs better than the RSF model because it produced the lowest values for the different sample sizes considered.

**Fig 5:**
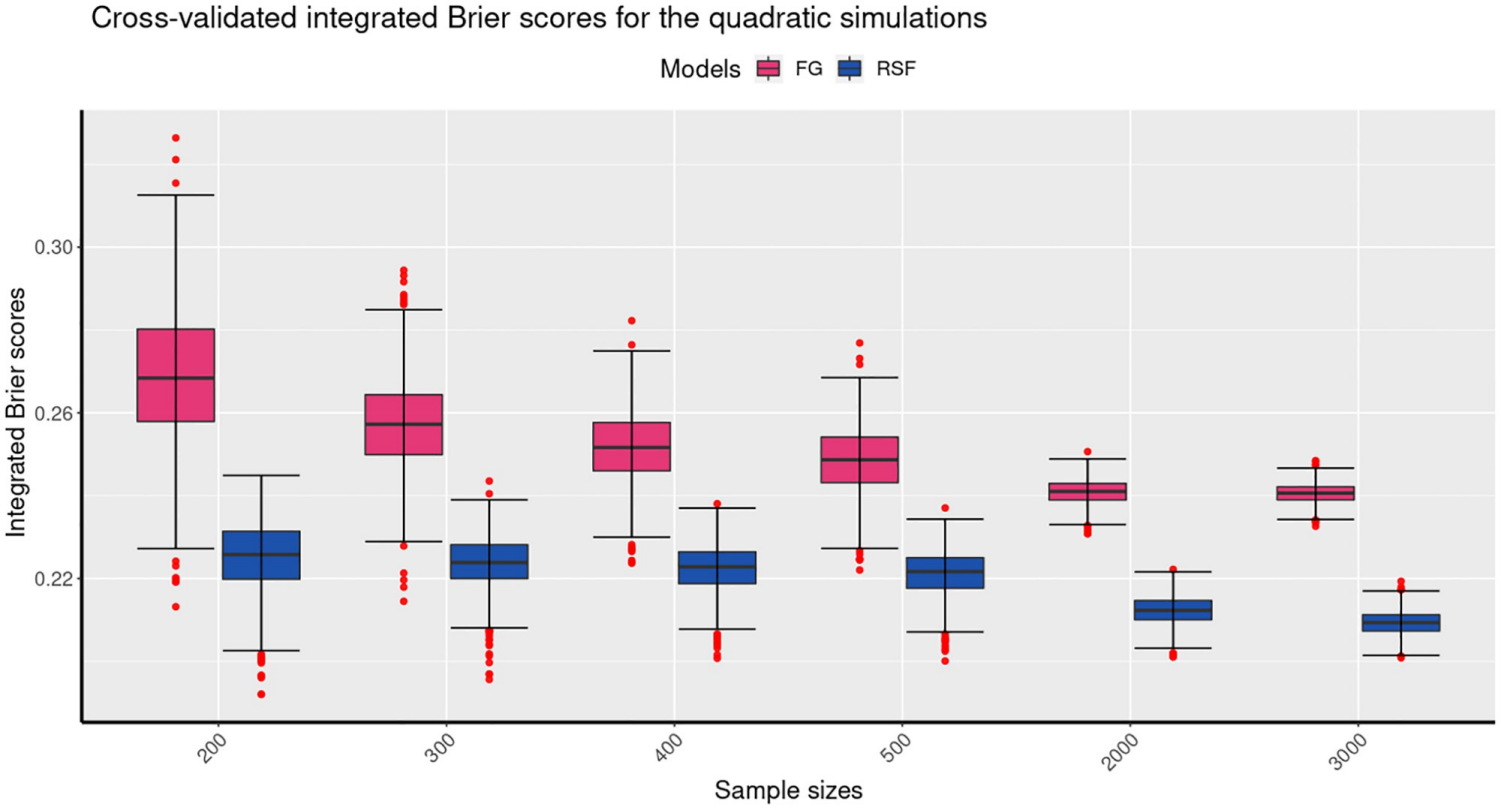
The boxplots present the mean cross-validated integrated brier scores from the 1000 simulations for each of the sample sizes for the quadratic simulations. The boxplots show the performance of the two models at six different sample sizes, 200, 300, 400, 500, 2000 and 3000. For the quadratic simulations, the RSF has lower mean IBS values compared to the FG model for the different sample sizes.

**Fig 6:**
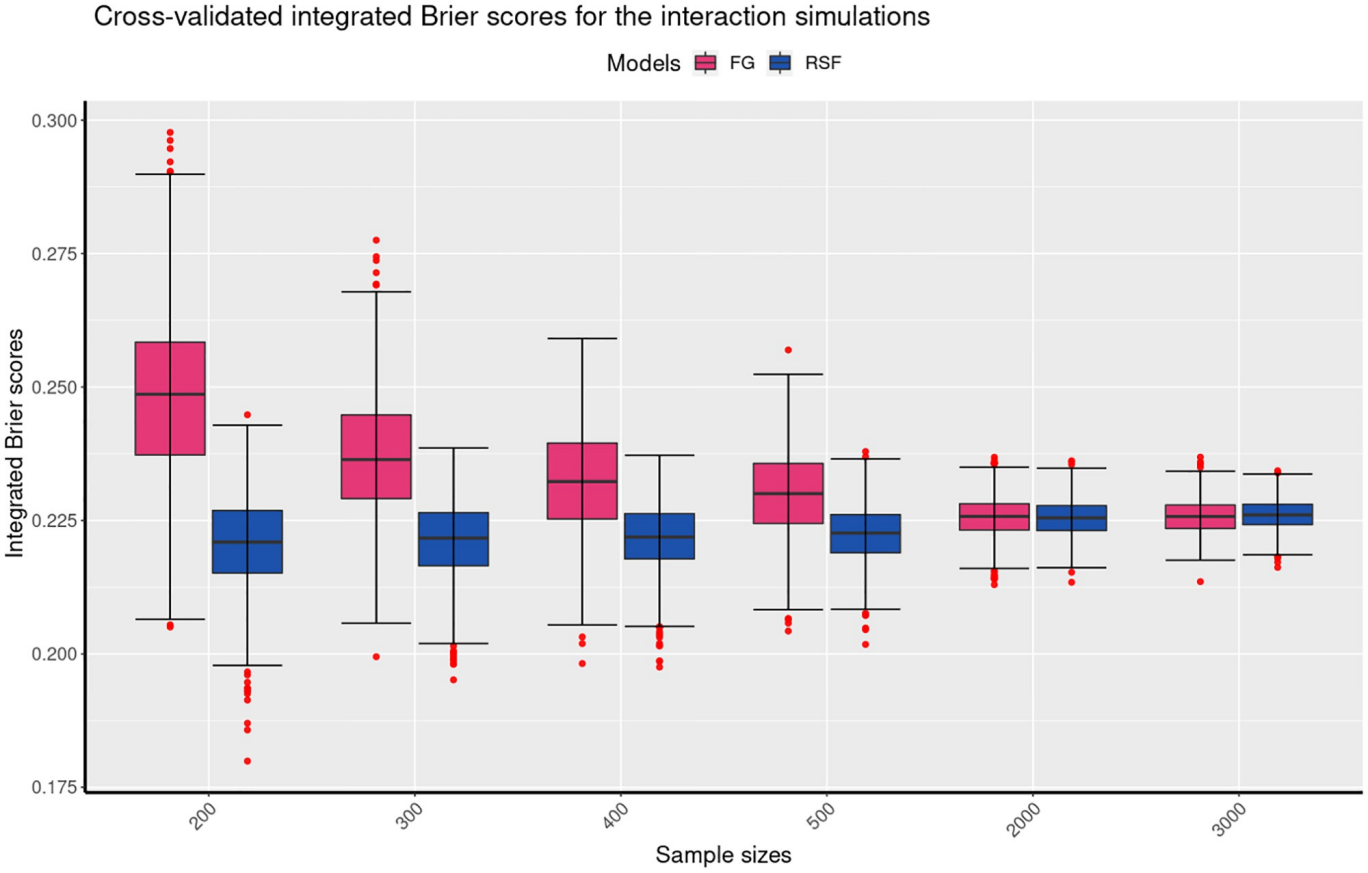
The boxplots present the mean cross-validated integrated Brier scores from the 1000 simulations for each of the sample sizes for the interaction simulations. The boxplots show the performance of the two models at six different sample sizes, 200, 300, 400, 500, 2000 and 3000. The boxplots show that the RSF model has lower mean cv IBS scores compared to the RSF model. Also, the variability in the predictive performance of the two models decreases with increasing sample sizes.

Figs 4 to 6 present a comparison of the mean cross-validated Integrated Brier Scores (IBS) for the linear, quadratic and interaction simulation results of the FG, and the RSF models. The results of the linear simulations are shown in Fig 4 indicate that for the different sample sizes, the mean cv IBS scores of the FG model are lower than those of the RSF model. The figure further shows that the mean cv IBS scores for the RSF model decrease markedly for larger sample sizes. These results therefore indicate that for the linear simulations, the FG outperforms the RSF model as it produced the lowest mean IBS for the different sample sizes. It is also important to note that the mean cv IBS scores are below 0.25, which indicates that both models are predictive on the datasets given. The results of the quadratic simulations are shown in Fig 5. They indicate that for different sample sizes, the mean cv IBS scores of the FG model are higher than those of the RSF model. In addition, Fig 5 shows that the mean cv IBS scores for the RSF model decrease with the increase in the sample size. These results show that for the quadratic simulations, the RSF outperforms the FG model as it produced the lowest mean IBS for the different sample sizes. Furthermore, Fig 5 shows that for the quadratic simulations, the IBS results of the RSF model are more consistent than those of the FG model. Fig 6 shows summarises of the results of the interaction simulations. The summary indicates that the RSF model has lower mean cv IBS scores compared to the RSF model. It is also important noted that for large samples (greater than 500) the results for the interaction simulations are indistinguishable. Figs 4 to 6 also show that the variability in the predictive performance of the two models decrease with increase in the sample size. Our results are consistent with previous studies that indicated that variability in predictive performance decreases with increasing sample size [35, 36]. Table 1 summarises all the simulations of the linear, quadratic and interaction results based on the FG and the RSF models. The results for the linear simulations show that the mean cv IBS scores for the FG model are on average between 0.16–0.18 across all sample sizes, which is lower compared to the mean cv IBS scores for the RSF model which are betwen 0.19–0.22 across all sample sizes.

**Table 1:** Summary of the simulations results.

Sample sizes	% of significant t-statistics	% of significant F-Statistics	IBS FG model	IBS RSF model
	Linear simulations			
200	20.10	8.80	0.18	0.22
300	38.40	21.60	0.17	0.21
400	47.90	32.60	0.17	0.21
500	60.10	43.10	0.16	0.21
2000	91.40	88.50	0.16	0.20
3000	94.90	95.40	0.16	0.19
	Interaction simulations			
200	21.40	10.20	0.25	0.22
300	16.80	6.70	0.24	0.22
400	15.00	5.70	0.23	0.22
500	12.80	3.30	0.23	0.22
2000	1.10	0.20	0.23	0.23
3000	1.80	0.10	0.23	0.23
	Quadratic simulations			
200	39.40	25.70	0.27	0.23
300	47.50	38.60	0.26	0.22
400	55.90	49.60	0.25	0.22
500	68.60	63.20	0.25	0.22
2000	99.90	99.90	0.24	0.21
3000	100.00	100.00	0.24	0.21

The mean CV IBS scores of the RSF and the FG model presented with the F and t-statics to show whether there is significant difference in performance for the two models.

The F and t-statistics show that the proportion of significant tests largely increases as the sample sizes increase for the linear simulations. This means that for larger sample sizes, the two models have an even large significant difference in their predictive performance. For example, up-to 95% of the simulated samples have significant F-statistics and t-statistics for the sample size *N* = 3000. The results imply that the FG model is superior in predictive performance in linear simulations compared to the RSF model because it has a lower mean IBS scores for linear simulations in larger sample sizes as shown in Table 1.

The results further show that, the mean cv IBS scores for the FG model on the quadratic simulations are on average between 0.24–0.27 across all sample sizes. In contrast, the mean cv IBS scores for the RSF model are on average much lower and between 0.21–0.23 across all sample sizes. The table shows that 100% of the samples considered have significant F and t-statistics for a sample size of *N* = 3000. This indicates that the performance of the RSF model on the quadratic simulations is statistically significant and better than that of the FG model especially in larger sample sizes.

The results in Table 1 also indicate that the mean cv IBS scores for the FG range from 0.23 to 0.25 for the interaction simulations compared to 0.22 to 0.23 for the RSF model. The results for the interaction simulations further suggest that this difference in the predictive performance of the two models is not statistically significant. This is because, the percentage of statistically significant t-statistics for the samples considered range from 1.80% to 21.40%. The percentage of significant F-statistics range from 0.10% to 10.20%. Large sample simulations confirm the result that the two model’s predictive performance is not significantly different with approximately 1.8% samples with significant t-statistics and 0.10% samples with significant F-statistics for the samples of size of *N* = 3000.

Table 1 states performance values together with the statistical tests results to tell whether these differences that exist in predictive performance are significant or not. This type of reporting is a good practice in clinical research especially when deciding on the best model to use when the choice is between a more interpretable (classical statistical) and a machine learning (black box) type of model. This is because the researcher can use the statistical test results to justify their model choice.

**Fig 7:**
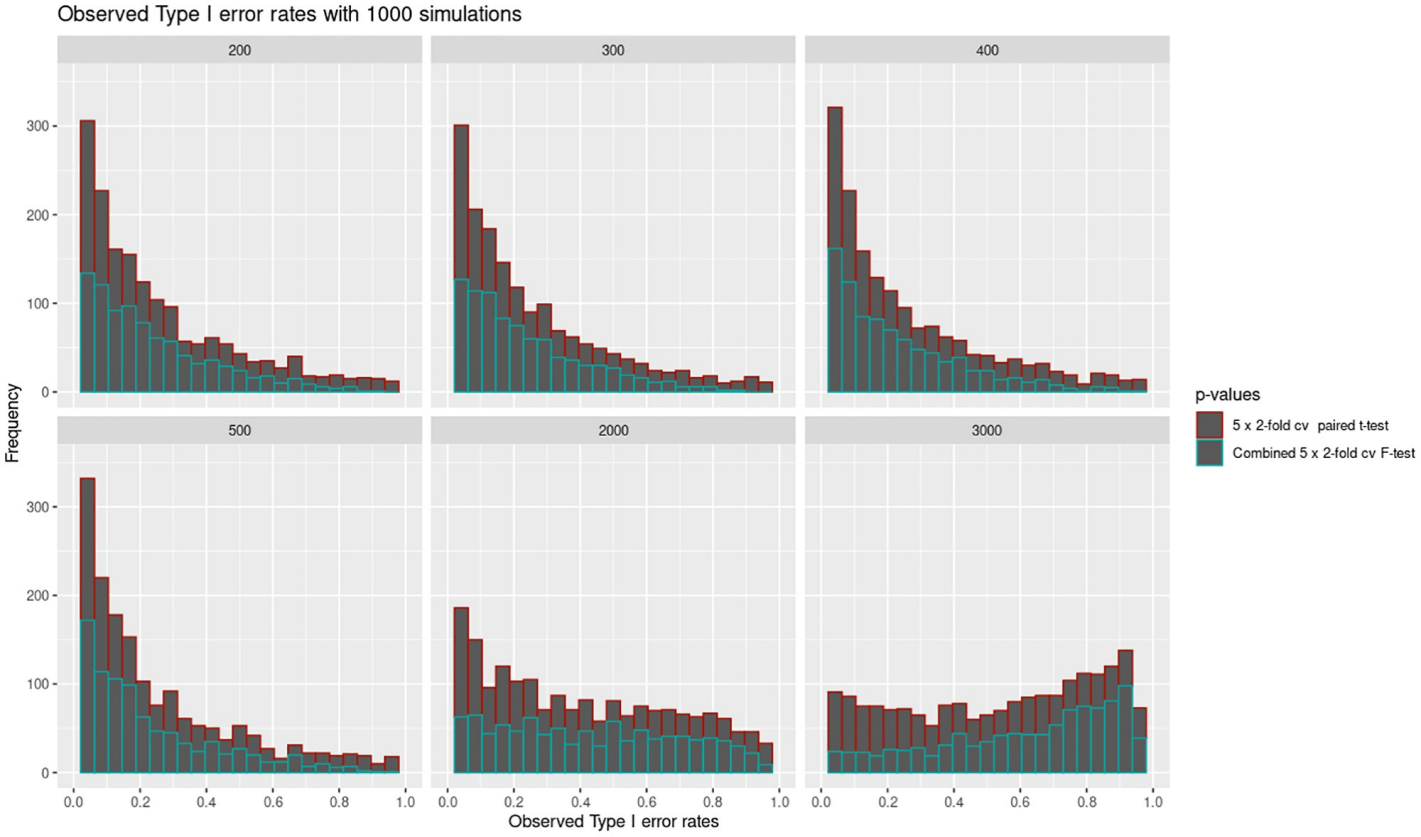
The histograms present the observed Type I error for the *5 × 2*-fold cv paired *t*-test, and the combined *5 × 2*-fold cv *F*-test under the null. The p-values for the two statistical tests under the null were obtained at six different sample sizes, 200, 300, 400, 500, 2000 and 3000. Under the null hypothesis, the *5 × 2*-fold cv paired *t*-test, has a higher Type I error compared to the combined *5 × 2*-fold cv *F*-test. The distribution for its observed *p*-values is uniform especially for larger sample sizes.

The study further investigated the Type I error of the *5 × 2*-fold cv paired *t*-test, and the combined *5 × 2*-fold cv *F*-test. Fig 7 shows that the *5 × 2*-fold cv paired *t*-test, has a higher Type I error compared to the combined *5 × 2*-fold cv *F*-test. This is expected because the combined *5 × 2*-fold cv *F*-test combines the results of the 10 possible statistics rather-than using only one of them. The observed *p*-values tend to have a uniform distribution for the larger sample sizes. The large type I error of the two tests in smaller sample sizes needs to be investigated further. However, the most plausible explanation of this phenomena arises from the assumptions made when constructing the tests. One of the assumptions is that the difference of two identically distributed predictive performance values ($p_{i}^{\left(j\right)}$) are assumed to be independent when in-fact they are not independent. The differences are also assumed to be independently normally distributed which is not strictly true because the training and test sets are not drawn independently of each other [14]. The strict assumption could be very amplified in the smaller sample sizes than in larger sample sizes. The independence assumption affects the combined *5 × 2*-fold cv *F*-test where the assumption is that $\;\sum_{i=1}^{5}\sum_{j=1}^{2}{\left(p_{i}^{\left(j\right)}\right)}^{2}\;$ and $\;\sum_{i=1}^{5}s_{i}^{2}\;$ are independent which is not technically true [14].

**Fig 8:**
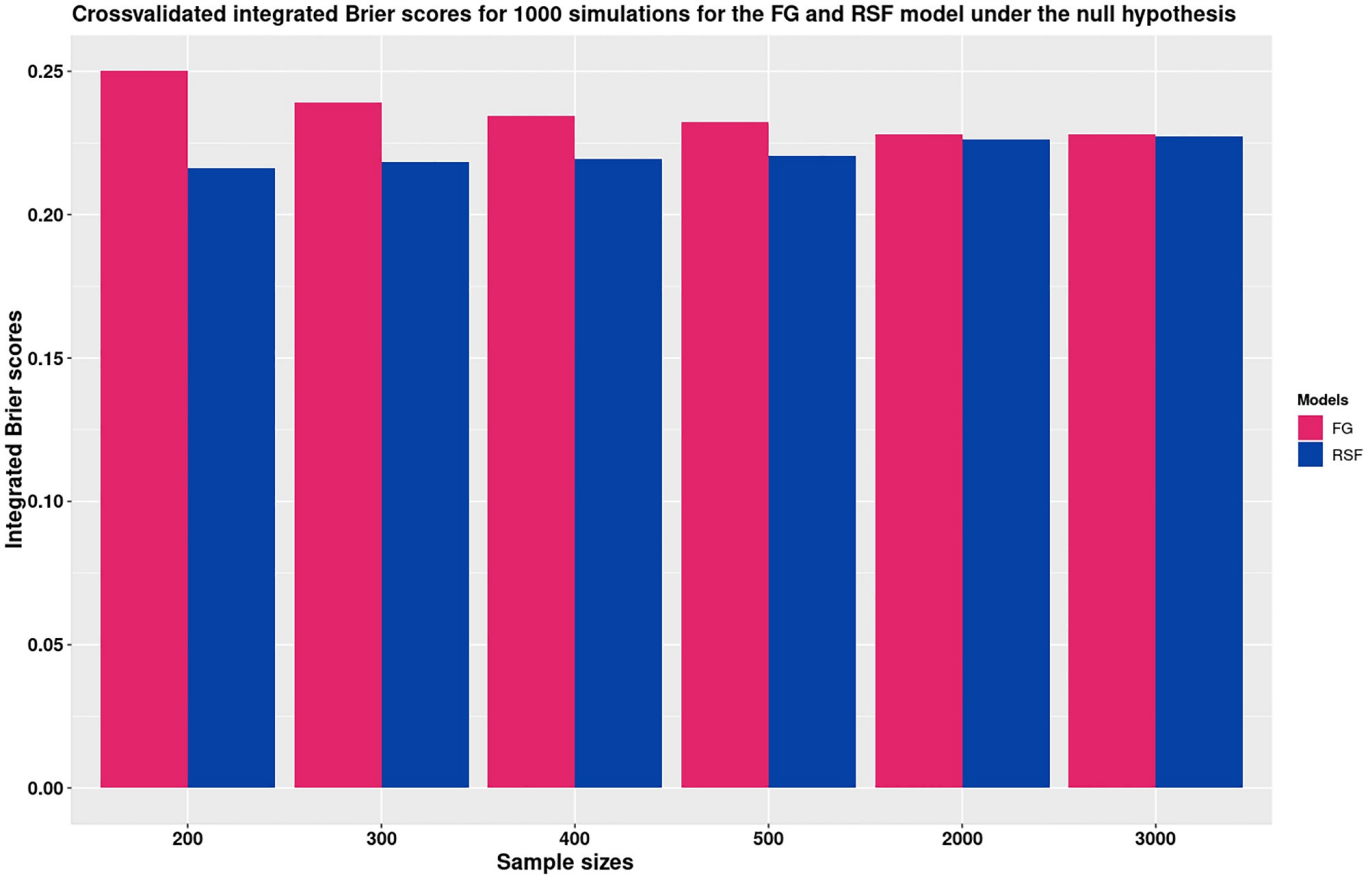
The bar charts compare the validation performance of the RSF and the FG model under the null hypothesis. Under the null hypothesis, the bar-charts are expected to have a height of 0.25. Under the null hypothesis, the bar charts show that the RSF model has smaller mean cv IBS values compared to the FG model for lower sample sizes. The charts also indicate that the RSF model has higher mean cv IBS as expected.

Another explanation is the fact that machine learning models trained on a small dataset are more likely to see patterns that do not exist, which results in high variance and very high error on a test set. These are the common signs of overfitting. A study by [37] used datasests to train supervised ML methods to classify healthy individuals and individuals with brain disorders. The study used datasets with smaller sample sizes with a median number of samples equal to 88 and interestingly, the overall reported accuracy was higher in the datasets with smaller sample sizes [37, 38]. A study by [38] trained machine learning and classical statistical methods using simulations at different sample sizes to provide an insight into whether the tendency to report higher performance estimates with smaller sample sizes could be due to insufficiently reliable validation. They used the K-Fold CV and their results showed that the machine learning model accuracies were considerably higher than the theoretical chance level of 50%. The highest difference was observed with smaller sample sizes; however, the difference was still evident even at the sample size of *N* = 1000. The results from these two studies agree with the results from our study as demonstrated in Fig 8 below. The RSF model which is a machine learning model has unexpected smaller IBS values compared to the FG model under the null hypothesis for smaller sample sizes. This implies that the RSF model was seeing patterns that did not exist as shown in Fig 8.

The bar charts in Fig 8 compare the validation performance of the RSF and the FG model under the null hypothesis. Under the null, the models are expected to have an IBS value of 0.25. The bar charts confirm that the RSF model had smaller mean cv IBS values especially for lower sample sizes compared to the FG model. The FG model model however, had higher mean cv IBS as expected under the null. That is to say, values close to 0.25.

**Fig 9:**
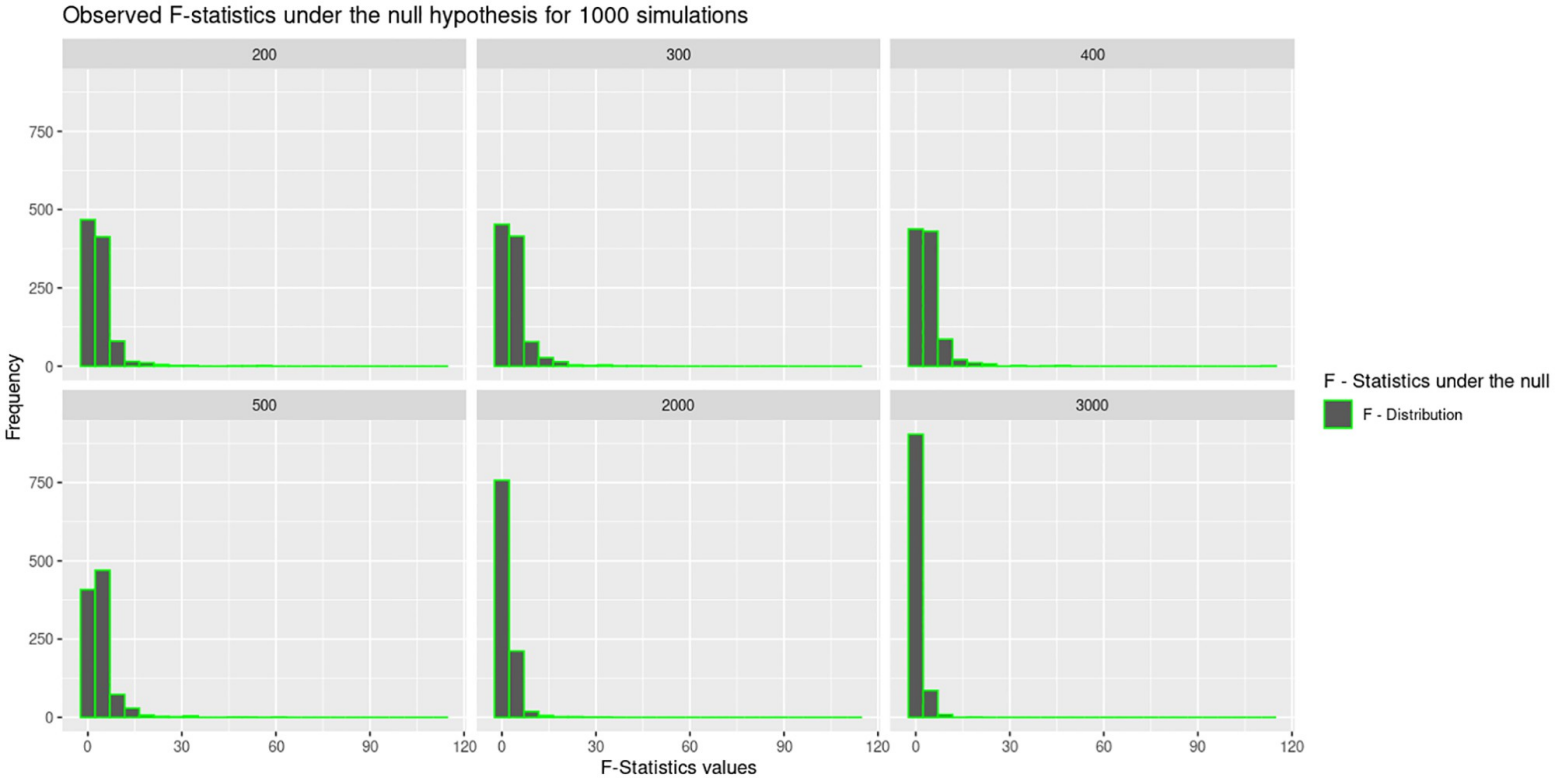
The histograms present the F statistics for the combined *5 × 2*-fold cv F test under the null at the six different sample sizes considered in this study. Under a true null hypothesis, the F-statistic is “hovering around” 1 after repeated computations.

**Fig 10:**
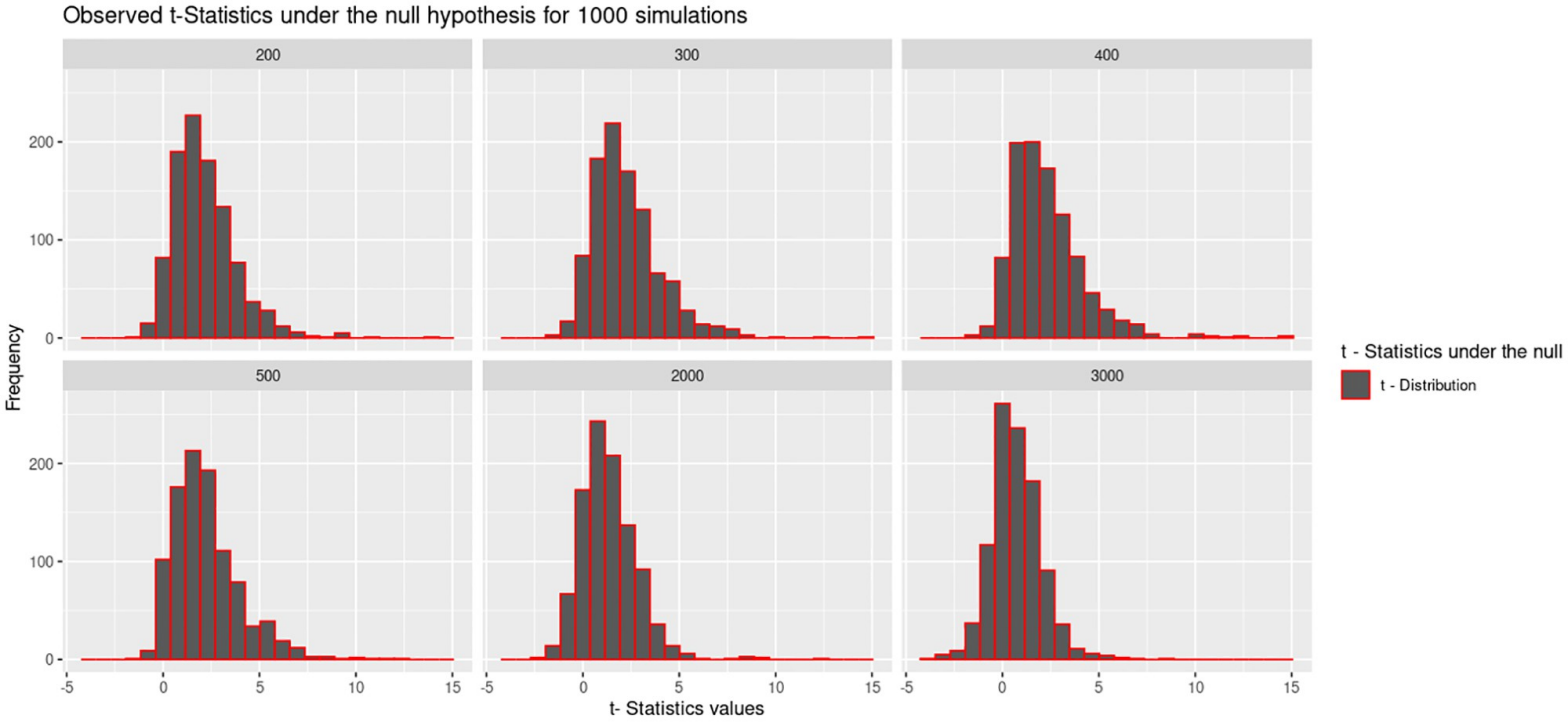
The histograms present the t statistics for the the *5 × 2*-fold cv paired t-test under the null at six different sample sizes considered in this study. Under the null, the t-statistics are approximately normally distributed, however, the peak of the graph is not at zero, for most of the sample sizes except for the largest sample size.

The study further investigated the distribution of the two test statistics under the null. The histograms in Figs 9 and 10, present the distribution of the these test statistics under the null hypothesis. Fig 9 shows that the F-statistics values are close to 1.0 for the large sample sizes *N* = 2000 and *N* = 3000.

This is an indication that the F-statistic is “hovering around” 1 after repeatedly computing the F-statistic for situations when the null is true. Fig 10 shows that the t-statistics under the null is normally distributed but the peak of the graph is not at zero, for most of the sample sizes except for the one where *N* = 3000. This indicates that obtaining a sample value close to the null hypothesis is most likely in a larger sample size.

## Discussion and conclusion

This study explores the existing statistical tests that can be used to identify a significant difference between classical and machine learning models in the analysis of survival data. The study trained two models, that is the Fine-Gray and the Random survival forests for competing risks in three low-dimensional data scenarios namely; the linear, the quadratic and the interaction models. The Fine-Gray is a classical statistical model while the random survival forest model for competing risks is a machine learning model. The study revealed that the FG model is superior in predictive performance in the linear-low-dimension data simulation scenarios and that the difference in the predictive performance in comparison to the RSF is significant.

The study further revealed that the RSF model has lower IBS values in the interaction low-dimension simulations but the two statistical tests showed that there is no significant difference in the predictive performance of this model in comparison with the FG model in the interaction simulations.

Furthermore, the study showed that the RSF model is superior in predictive performance in quadratic low-dimension data simulation scenarios compared to the FG model. The F and the t-statistics tests also revealed that the difference in this predictive performance is highly significant especially in large data samples.

This study confirms that in the presence of complex relationships between the outcome and the predictors, the machine learning model (RSF) is superior in predictive performance. In linear simulations, however, the FG model model is superior. These results are similar to those obtained in the study by [5]. However, this study goes further to state whether this difference in predictive performance is significant or not.

The results revealed that sometimes there is no significant difference between the classical statistical model and the machine learning model. Having knowledge of this can guide clinicians to use the most interpretable model. This result is important especially if the goal of any given study is not to predict the outcome which is usually the motivation for using a machine learning model.

The study recommends that statistical tests such as the ones used in this study that is, *5 × 2*-fold cv paired *t*-test, and the combined *5 × 2*-fold cv *F*-test become part of regular practice in justification for the use of both machine learning and classical statistics models for data analysis in medical studies.

## References

[1] Qin SJ, Chiang LH. Advances and opportunities in machine learning for process data analytics. Computers & Chemical Engineering. 2019;126:465–473. doi: 10.1016/j.compchemeng.2019.04.003

[2] Goodfellow IJ, Erhan D, Carrier PL, Courville A, Mirza M, Hamner B, et al. Challenges in representation learning: A report on three machine learning contests. In: International conference on neural information processing. Springer; 2013. p. 117–124.

[3] Ghahramani Z. Probabilistic machine learning and artificial intelligence. Nature. 2015;521(7553):452–459. doi: 10.1038/nature14541 26017444

[4] Fine JP, Gray RJ. A proportional hazards model for the subdistribution of a competing risk. Journal of the American Statistical Association. 1999;94(446):496–509. doi: 10.1080/01621459.1999.10474144

[5] Ishwaran H, Gerds TA, Kogalur UB, Moore RD, Gange SJ, Lau BM. Random survival forests for competing risks. Biostatistics. 2014;15(4):757–773. doi: 10.1093/biostatistics/kxu010 24728979 PMC4173102

[6] Lin D. Non-parametric inference for cumulative incidence functions in competing risks studies. Statistics in Medicine. 1997;16(8):901–910. doi: 10.1002/(SICI)1097-0258(19970430)16:8<901::AID-SIM543>3.0.CO;2-M 9160487

[7] Blumenstock Gabriel and Lessmann Stefan and Seow Hsin-Vonn. Deep learning for survival and competing risk modelling. Journal of the Operational Research Society. 2022;73(1):26–38. doi: 10.1080/01605682.2020.1838960

[8] Misaii Hasan and Eftekhari Mahabadi Samaneh and Haghighi Firoozeh. Multiple imputation of masked competing risks data using machine learning algorithms. Journal of Statistical Computation and Simulation. 2022;1–26.

[9] Zhang X, Akcin H, Lim HJ. Regression analysis of competing risks data via semi-parametric additive hazard model. Statistical Methods & Applications. 2011;20(3):357–381. doi: 10.1007/s10260-011-0161-4

[10] Austin PC, Fine JP. Practical recommendations for reporting F ine-G ray model analyses for competing risk data. Statistics in Medicine. 2017;36(27):4391–4400. doi: 10.1002/sim.7501 28913837 PMC5698744

[11] Austin Peter C and Steyerberg Ewout W. The Integrated Calibration Index (ICI) and related metrics for quantifying the calibration of logistic regression models. Statistics in medicine. 2019;38(21):4051–4065. doi: 10.1002/sim.8281 31270850 PMC6771733

[12] Austin Peter C and Putter Hein and Giardiello Daniele and van Klaveren David. Graphical calibration curves and the integrated calibration index (ICI) for competing risk models. Diagnostic and prognostic research. 2022;6(1):1–22. doi: 10.1186/s41512-021-00114-6 35039069 PMC8762819

[13] Dietterich TG. Approximate statistical tests for comparing supervised classification learning algorithms. Neural Computation;. 9744903 10.1162/089976698300017197

[14] Alpaydm E. Combined 5 × 2 cv F test for comparing supervised classification learning algorithms. Neural Computation. 1999;11(8):1885–1892. doi: 10.1162/089976699300016007 10578036

[15] Lee S, Lim H. Review of statistical methods for survival analysis using genomic data. Genomics & Informatics. 2019;17(4). doi: 10.5808/GI.2019.17.4.e41 31896241 PMC6944043

[16] Wang P, Li Y, Reddy CK. Machine learning for survival analysis: A survey. ACM Computing Surveys (CSUR). 2019;51(6):1–36. doi: 10.1145/3214306

[17] Deo SV, Deo V, Sundaram V. Survival analysis—part 1. Indian Journal of Thoracic and Cardiovascular Surgery. 2020;36(6):668–672. doi: 10.1007/s12055-020-01049-1 33100633 PMC7572944

[18] Lee ET, Wang J. Statistical methods for survival data analysis. vol. 476. John Wiley & Sons; 2003.

[19] Mohammad KA, Fatima-Tuz-Zahura M, Bari W. Fine and Gray competing risk regression model to study the cause-specific under-five child mortality in Bangladesh. BMC International Health and Human Rights. 2017;17(1):1–8. doi: 10.1186/s12914-017-0112-8 28129793 PMC5273814

[20] Cox DR. Regression models and life-tables. Journal of the Royal Statistical Society Series B (Methodological). 1972;34:187–220. doi: 10.1111/j.2517-6161.1972.tb00899.x

[21] Austin PC, Lee DS, Fine JP. Introduction to the analysis of survival data in the presence of competing risks. Circulation. 2016;133(6):601–609. doi: 10.1161/CIRCULATIONAHA.115.017719 26858290 PMC4741409

[22] Coviello V, Boggess M. Cumulative incidence estimation in the presence of competing risks. The Stata Journal. 2004;4(2):103–112. doi: 10.1177/1536867X0400400201

[23] Buzkova P. Competing risk of mortality in association studies of non-fatal events. Plos One. 2021;16(8):e0255313. doi: 10.1371/journal.pone.0255313 34388170 PMC8362942

[24] Nasejje JB, Mwambi H, Dheda K, Lesosky M. A comparison of the conditional inference survival forest model to random survival forests based on a simulation study as well as on two applications with time-to-event data. BMC Medical Research Methodology. 2017;17(1):1–17. doi: 10.1186/s12874-017-0383-8 28754093 PMC5534080

[25] Breiman L. Random forests. Machine Learning. 2001;45(1):5–32. doi: 10.1023/A:1010933404324

[26] Ishwaran H, Kogalur UB, Blackstone EH, Lauer MS. Random survival forests. The Annals of Applied Statistics. 2008; p. 841–860.

[27] Ishwaran H, Kogalur UB. randomForestSRC: Random Forests for Survival, Regression and Classification (RF-SRC). R Package Version. 2014;.

[28] Zhang MJ, Zhang X, Scheike TH. Modeling cumulative incidence function for competing risks data. Expert Review of Clinical Pharmacology. 2008;1(3):391–400. doi: 10.1586/17512433.1.3.391 19829754 PMC2760993

[29] Segal MR. Regression trees for censored data. Biometrics. 1988;44:35–47. doi: 10.2307/2531894

[30] Beyersmann J, Latouche A, Buchholz A, Schumacher M. Simulating competing risks data in survival analysis. Statistics in Medicine. 2009;28(6):956–971. doi: 10.1002/sim.3516 19125387

[31] Ishwaran H, Kogalur UB, Kogalur MUB. Package â[U+0080][U+0098] randomForestSRCâ[U+0080][U+0099]. Breast. 2022;6:1.

[32] Gray B, Gray MB, Gray R. The cmprsk package. The Comprehensive R Archive Network. 2004;.

[33] Brier GW. Verification of forecasts expressed in terms of probability. Monthly Weather Review. 1950;78(1):1–3. doi: 10.1175/1520-0493(1950)078<0001:VOFEIT>2.0.CO;2

[34] Haider H, Hoehn B, Davis S, Greiner R. Effective ways to build and evaluate individual survival distributions. J Mach Learn Res. 2020;21(85):1–63.34305477

[35] Cumming GS. Using between-model comparisons to fine-tune linear models of species ranges. Journal of Biogeography. 2000;27(2):441–455. doi: 10.1046/j.1365-2699.2000.00408.x

[36] Wisz MS, Hijmans R, Li J, Peterson AT, Graham C, Guisan A, et al. Effects of sample size on the performance of species distribution models. Diversity and Distributions. 2008;14(5):763–773. doi: 10.1111/j.1472-4642.2008.00482.x

[37] Yu C, Li J, Liu Y, Qin W, Li Y, Shu N, et al. White matter tract integrity and intelligence in patients with mental retardation and healthy adults. Neuroimage. 2008;40(4):1533–1541. doi: 10.1016/j.neuroimage.2008.01.063 18353685

[38] Vabalas A, Gowen E, Poliakoff E, Casson AJ. Machine learning algorithm validation with a limited sample size. Plos One. 2019;14(11):e0224365. doi: 10.1371/journal.pone.0224365 31697686 PMC6837442

